# Rabies Acquired through Mucosal Exposure, China, 2013 

**DOI:** 10.3201/eid2505.181413

**Published:** 2019-05

**Authors:** Hong Zhao, Jian Zhang, Cong Cheng, Yi-Hua Zhou

**Affiliations:** Second Hospital of Nanjing at Southeast University, Nanjing, China (H. Zhao, J. Zhang, C. Cheng);; Nanjing University Medical School, Nanjing (Y.-H. Zhou)

**Keywords:** rabies, mucosal exposure, postexposure prophylaxis, vaccine, viruses, China

## Abstract

In China in 2013, a man acquired rabies after sucking wounds of his son, who had been bitten by a stray dog. The man declined postexposure prophylaxis (hyperimmunoglobulin and vaccine) and died; the son accepted prophylaxis and survived. Physicians should be aware of rabies transmission through mucosal exposure and encourage postexposure prophylaxis.

Rabies, caused by the rabies virus (family *Rhabdoviridae*, genus *Lyssavirus*), is a zoonotic encephalitis with a mortality rate of almost 100%. Although rare in industrialized countries because of mandatory vaccination of dogs and other domestic pets, rabies frequently occurs in developing countries (*1*). In industrialized countries, the virus is transmitted to humans mostly by contact with wild animals; in developing countries, by dog bites (*1*). We report a fatal case of rabies in a man who had sucked the wounds of his son who had been bitten by a stray dog. The study was approved by the institutional ethics review committees of the Second Hospital of Nanjing and Nanjing Drum Tower Hospital, Nanjing, China, and was performed in accordance with the ethics standards in the 1964 Declaration of Helsinki and its later amendments. Written informed consent was obtained from the patient’s son.

The patient was a 41-year-old man from a village in northern Jiangsu Province, China. On August 19, 2013, the man’s 17-year-old son was bitten on the left leg by a stray dog, leaving bleeding wounds on the lower part of the gastrocnemius muscles. Because of concern about rabies, the man and other family members immediately washed the boy’s wounds with water, and the man’s neighbors killed and buried the stray dog. While washing his son’s wounds, the man made a dramatic scene by crouching down to suck “toxic blood” from his son’s wounds and spitting out the blood several times. He then sent his son to a local hospital, where the physicians immediately (within 1 hour after the dog bite) cleaned the wounds by using standard procedures and administered postexposure prophylaxis (rabies hyperimmunoglobulin and the first dose of freeze-dried rabies vaccine for human use [Vero cells]; Liaoning Chengda Biotechnology, http://www.cdbio.cn). The son received another 4 doses of rabies vaccine on days 3, 7, 14, and 28 after the bite. Because the man had sucked the wounds, the physicians also recommended that he receive postexposure prophylaxis (rabies hyperimmunoglobulin and vaccine); however, the man declined because of concern over the relatively high cost and because he considered that he had spat out all the sucked blood and could not have “so bad luck.” Thus, the man did not receive any medical treatment.

On September 23, 2013, the man was referred to the Nanjing Second Hospital, the main infectious diseases hospital in Jiangsu, with a 2-day history of general malaise, sleep disturbance, waking in a cold sweat, irritability to air and sound, involuntary contraction of pharyngeal muscles, and choking when drinking and eating. At the time of examination, the patient showed remarkable agitation, delirium, and hallucination. Saliva was collected and tested for rabies viral RNA by reverse transcription PCR; the result was positive for rabies virus. The patient received systemic support and symptomatic therapy but died on September 24. The clinical course of rabies in this patient progressed rapidly; although the incubation period for this patient was 33 days (in agreement with incubation period of 20–60 days for most patients), he died 3 days after the appearance of furious (classical) symptoms. The patient’s son remained in good health 5 years later.

The main route for rabies virus transmission is bites of infected dogs and other animals. Unusual transmission routes include mucosal exposure, aerosol inhalation, organ transplantation (*2*–*7*), and contamination of broken skin by blood from wounds of a person bitten by an infected dog (*8*). The most effective way to control rabies in humans is mandatory vaccination of all dogs and other domestic pets, but widespread vaccination is not feasible in China and resource-limited countries. Although China’s central and local governments recommend that all domestic pets be vaccinated against rabies, implementation of vaccination is difficult in many rural areas of China. Thus, for preventing rabies in humans in rabies-endemic countries and regions, postexposure prophylaxis is crucial. 

The rabies case that we report was acquired after sucking wounds created by the bite of a presumably rabies-infected dog. Evidently, the virus invaded the patient through his oral mucosa. Unfortunately, the patient declined postexposure prophylaxis and died. 

This case underscores the possibility of rabies transmission by mucosal exposure, which should be made widely known to the public. To further reduce this disease in rabies-endemic countries, physicians should emphasize the high likelihood of transmission of rabies virus after mucosal exposure and try to persuade persons at risk to receive postexposure prophylaxis. 
